# Histologic and molecular characterization of a *MAZ::NCOA2* fusion-positive intracranial neoplasm

**DOI:** 10.17879/freeneuropathology-2025-9012

**Published:** 2025-11-11

**Authors:** Elliot Stalter, Claire Voyles, Leonardo F. Freitas, Martha M. Quezado, Brian J. Dlouhy, Andrew Groves, Osorio Lopes Abath Neto

**Affiliations:** 1 University of Iowa Carver College of Medicine, Iowa City, USA; 2 Florida International University, Herbert Wertheim College of Medicine, Miami, USA; 3 Laboratory of Pathology, Center for Cancer Research, National Cancer Institute, National Institutes of Health, Bethesda, USA; 4 Department of Neurosurgery, University of Iowa Health Care, Iowa City, USA; 5 Department of Pediatrics, University of Iowa Health Care, Iowa City, USA; 6 Department of Pathology, Neuropathology, University of Iowa Health Care, Iowa City, USA

**Keywords:** Infantile CNS tumor, Gene fusion, MAZ::NCOA2, Glioma, Case report

 Gene fusions have emerged as one of the primary drivers of oncogenesis in various tumor types, functioning through activation of signaling pathways or dysregulation of transcription. More than one third of soft tissue tumor types harbor gene fusions, over half of which are recurrent, and in the central nervous system (CNS) fusions are uniquely associated with infantile tumors^1^. Identification of novel fusions is essential to map the landscape of diagnostic tumor types and to develop targeted therapies. Here we describe the first reported case of an intracranial neoplasm harboring a *MAZ::NCOA2* fusion, highlighting a novel genetic alteration in the central nervous system and expanding the molecular spectrum of infantile fusion-driven neoplasms. 

A previously healthy 12-month-old female presented with a 3-week history of developmental regression, intermittent seizures, and truncal ataxia. Physical examination revealed hypotonia, generalized hyporeflexia, and a head circumference greater than the 99^th^ percentile for age. Brain imaging demonstrated a large left parieto-occipital intraparenchymal mass with cystic and solid components, heterogeneous contrast enhancement, and signs of hydrocephalus with uncal herniation secondary to mass effect (**Figure 1A**). There were small areas of meningeal contact in the mesial left parieto-occipital regions, but no meningeal thickening or enhancement were present. Urgent craniotomy was performed with successful gross total resection of the mass (**Figure 1B**). Staging procedures were performed, and tumor was determined to be localized based on negative spine MRI and clear CSF on lumbar puncture (performed > 14 days after resection).

**Figure 1:**
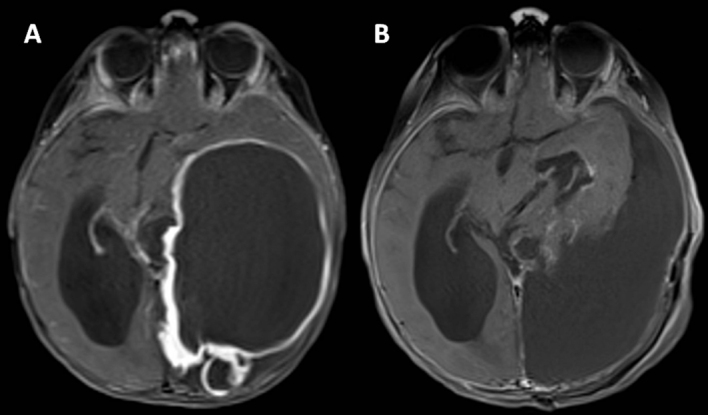
**A.** Brain MRI (post-contrast axial T1-weighted) revealed a contrast-enhancing left parieto-occipital mass (11.5 x 7.4 x 8.2 cm) with mixed cystic and solid components. The tumor abuts the leptomeninges but there is no meningeal thickening or enhancement. There is secondary hydrocephalus and midline shift resulting from the mass effect. **B.** Post-operative imaging (post-contrast axial T1-weighted) showing gross total resection of the mass.

Histologically, the neoplasm was well-circumscribed (**Figure 2A, 2B**) and showed variable cellularity. Regions of lower cellularity predominantly consisted of spindle cells with a fibrillary cytoplasm (**Figure 2C**) embedded in a heterogeneous myxoid stroma, which included frequent cystic spaces containing basophilic material (**Figure 2D**). Regions of higher cellularity (**Figure 2E**) were located at the interface with brain parenchyma and displayed elevated mitotic activity, focal necrosis, and Ki-67 proliferative rates of up to 30 % (**Figure 2L**). The immunophenotype of the neoplastic cells followed a regional distribution, with diffuse strong GFAP positivity (**Figure 2F**) but mostly absent OLIG2 expression (**Figure 2H**) in the lower-cellularity regions, but loss of GFAP expression (**Figure 2G**) and focal strong OLIG2 positivity (**Figure 2I**) in the higher-cellularity regions. SOX10 expression was strong and diffuse (**Figure 2J**), and CD99 was weak but diffuse (**Figure 2K**). Neurofilament decorated rare cells within the tumor. Markers that were notably negative in the neoplastic population included synaptophysin, CD34, EMA, desmin, myogenin, NKX2.2, pan-keratin, smooth muscle actin, p63, p40, ALK, ROS1, BRAF V600E, HEY1, and BCOR. Nuclear expressions of INI1, BRG1, ATRX, and H3 K27me3 were retained, and p53 had a non-clonal staining pattern. Beta-catenin showed cytoplasmic positivity but only equivocal weak nuclear staining in rare cells.

**Figure 2:**
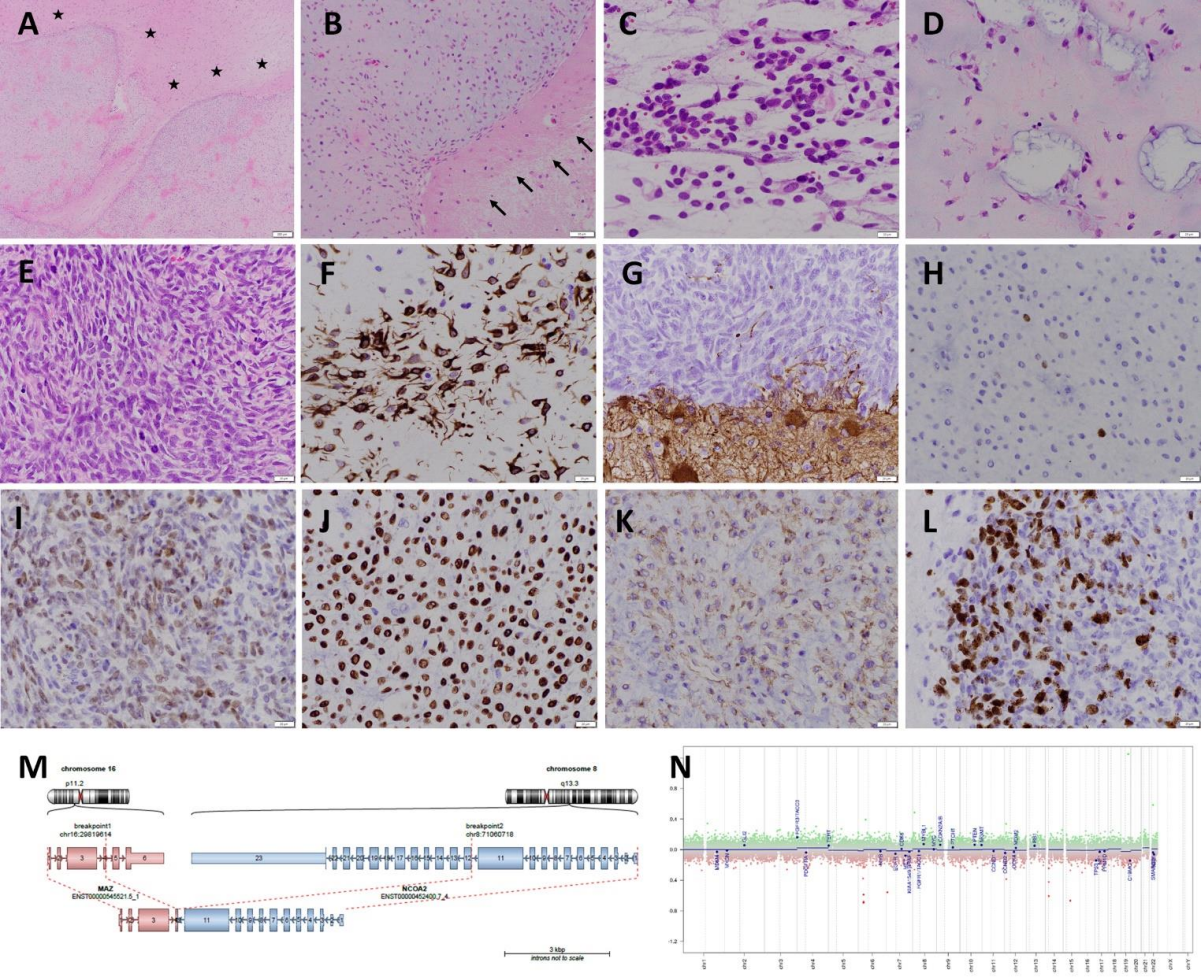
Morphologic and molecular features of the *MAZ::NCO2* fusion-positive tumor. **A–B.** The neoplasm (**A**; H&E, 100X) is well demarcated from the surrounding reactive brain parenchyma (asterisks), which contains numerous gemistocytic astrocytes and a rim of gliosis indicated by arrows (**B;** H&E, 200X). **C–L. **An intraoperative smear preparation (**C;** H&E, 400X) shows that tumor cells are spindled, with hyperchromatic elongated nuclei and processes. The tumor architecture varies from areas of relatively lower cellularity with a loose myxoid and cystic background (**D;** H&E, 400X) to areas of marked hypercellularity and mitotic activity (**E;** H&E, 400X). GFAP staining (**F, G;** 400X) shows positivity in tumor cells in looser areas (**F**) but loss of expression in more cellular areas (**G,** top), in contrast to the reactive brain parenchyma (bottom). OLIG2 (**H, I;** 400X), by contrast, is mostly negative in looser areas (**H**) but positive in areas of higher cellularity (**I**). Notable markers for which tumor cells are positive include SOX10 (**J, **400X) and CD99 (**K,** 400X). The Ki-67 proliferative rate (**L;** 400X) is elevated in hypercellular areas, up to 30 %. **M. **In-frame *MAZ::NCOA2* fusion including exon 4 of *MAZ* and exon 12 of *NCOA2*. **N.** Copy number plot extracted from DNA methylation array showed a flat profile. Clicking into the respective picture will lead you to the full virtual slide https://doi.org/10.57860/min_dts_000025

 Molecular testing was performed using customized targeted next-generation sequencing (NGS) panels for mutations and fusions using an Illumina NextSeq platform at University of Iowa. The DNA panel covers the full coding sequence of 93 and hotspot mutation regions of 109 cancer-related genes, while the RNA fusion panel can interrogate known and novel fusions involving 146 cancer-related genes. Testing detected an in-frame *MAZ::NCOA2* fusion involving the DNA-binding domain of *MAZ* (ENST00000545521.5_1) at exon 4 and the transactivation domain of *NCOA2* (ENST00000452400.7_4) at exon 12 (**Figure 2M**). A pathogenic missense mutation in *CTNNB1* (c.134C>T, p.Ser45Phe) was also detected, despite the inconclusive beta-catenin immunostain. DNA methylation profiling did not result in a match using the German Cancer Research Center (DKFZ) CNS tumor classifier versions 11b6 and 12.8, DKFZ sarcoma classifier version 12b6, or National Cancer Institute Bethesda classifiers versions 2 and 3. Highest scoring methylation classes were divergent for the various classifiers and all resulted in very low scores: "CNS tumor with PATZ1 fusion" (score 0.229) and "Ewing sarcoma" (score 0.222) for Bethesda v3; "chordoma" (score 0.108) and "hemangioblastoma" (score 0.04) for DKFZ CNS v12.8; and "osteosarcoma" (score 0.163) and "chondrosarcoma" (score 0.07) for DKFZ sarcoma v12b6. Dimensionality reduction techniques (UMAP and t-SNE) likewise did not show consistent clustering in the embedding. Copy number plots derived from methylation data revealed a flat profile, consistent with a stable genome (**Figure 2N**). These findings supported a *MAZ::NCOA2* fusion-positive neoplasm, not elsewhere classified. The patient was treated as per POG9233 ("Baby POG") with multi-agent chemotherapy including vincristine, cyclophosphamide, cisplatin, and etoposide for 72 weeks, without radiation therapy^2^. She developed cisplatin-related hearing loss but remains without evidence of disease 22 months after resection. 

 The *NCOA2* gene is a transcriptional coactivator in the p160 steroid receptor coactivator family, playing a role in the regulation of muscle differentiation^3^. *NCOA2* rearrangements have been described in several soft tissue tumor types, including congenital spindle cell rhabdomyosarcoma, mesenchymal chondrosarcoma, angiofibroma of soft tissue, myoepithelioma, and vascular tumors. *MAZ* encodes the MYC-associated zinc finger protein, a broadly expressed transcription factor implicated in multiple cell programs that binds to GC-rich DNA motifs and regulates the *MYC* promoter and other regions^4,5^. 

 A *MAZ::NCOA2* fusion has been previously reported in a case of intraorbital myoepithelioma^6^, and recently it has been identified in a subcutaneous round cell sarcoma in a 10-month-old^7^. However, it has never been described in CNS tumors, expanding the spectrum of *NCOA2*-rearranged tumors. The morphology of our case is neither reminiscent of myoepithelioma nor small round cell sarcoma, but there is some immunophenotypic overlap, particularly the positivity for SOX10. The absence of a match in DNA methylation classifiers suggests *MAZ::NCOA2* fusion-positive tumors may represent an as yet unrecognized CNS tumor type. Comparisons of methylation profiling of reported cases and identification of additional examples are important next steps to further characterize these tumors. 

From a clinical standpoint, the significance of the fusion is unclear. *NCOA2* fusion-positive tumors in pediatric soft tissues can show variable outcomes, with biologic behaviors that vary from indolent to aggressive. Our patient remains disease free at 22 months after gross total resection and chemotherapy, but longer follow-up and additional reported cases will be necessary to firmly establish outcomes.

## Conflict of interest statement

The authors report no conflicts of interest.

## Supplementary material

Supplementary File 1. Supplementary Material 1-6 (PDF file, 1270 KB):

- Supplementary Figure 1: Beta-catenin stain
- Supplementary Figure 2: p40 stain
- Supplementary Figure 3: pan-keratin stain
- Supplementary Figure 4: SMA stain
- Supplementary Figure 5: t-SNE Bethesda classifier v31
- Supplementary Figure 6: UMAP Bethesda classifier

## Supplementary Material

Supplementary File 1. Supplementary Material 1-6 (PDF file, 1270 KB)

## References

[R1] Mertens F, Antonescu CR, Mitelman F. Gene fusions in soft tissue tumors: Recurrent and overlapping pathogenetic themes. Genes Chromosomes Cancer 2016;55(4):291-310. 10.1002/gcc.22335 PMC5012284 26684580

[R2] Strother DR, Lafay-Cousin L, Boyett JM, Burger P, Aronin P, Constine L, et al. Benefit from prolonged dose-intensive chemotherapy for infants with malignant brain tumors is restricted to patients with ependymoma: a report of the Pediatric Oncology Group randomized controlled trial 9233/34. Neuro Oncol 2014 Mar;16(3):457-65. 10.1093/neuonc/not163 PMC3922508 24335695

[R3] Mosquera JM, Sboner A, Zhang L, Kitabayashi N, Chen CL, Sung YS, et al. Recurrent NCOA2 gene rearrangements in congenital/infantile spindle cell rhabdomyosarcoma. Genes Chromosomes Cancer 2013;52(6):538-50. 10.1002/gcc.22050. PMC3734530 23463663

[R4] Bossone SA, Asselin C, Patel AJ, Marcu KB. MAZ, a zinc finger protein, binds to c-MYC and C2 gene sequences regulating transcriptional initiation and termination. Proc Natl Acad Sci U S A 1992;89(16):7452-6. 10.1073/pnas.89.16.7452 PMC49728 1502157

[R5] Song J, Murakami H, Tsutsui H, Tang X, Matsumura M, Itakura K, et al. Genomic organization and expression of a human gene for Myc-associated zinc finger protein (MAZ). J Biol Chem 1998;273(32):20603-14. 10.1074/jbc.273.32.20603 9685418

[R6] An S, Koh HH, Chang ES, Choi J, Song JY, Lee MS, et al. Unearthing novel fusions as therapeutic targets in solid tumors using targeted RNA sequencing. Front Oncol 2022;12:892918. 10.3389/fonc.2022.892918 PMC9399837 36033527

[R7] Chen H, Zhang P, Zhou L. A MAZ::NCOA2 subcutaneous small round cell sarcoma of infancy with diffuse S100/SOX10 positivity: a novel entity. Genes Chromosomes Cancer 2025;64(2):e70034. 10.1002/gcc.70034 39985329

