# Supplementary material for: Histologic and molecular characterization of a MAZ::NCOA2 fusion-positive intracranial neoplasm
Source: Free Neuropathol. 2025 Nov 11;6:21. doi: 10.17879/freeneuropathology-2025-9012 (PMC12612709; doi:10.17879/freeneuropathology-2025-9012)
Supplement: Supplementary file 1 [file freeneuropathol-06-21-9012-s1.pdf]

# Supplementary material

- **Supplementary Figure 1: Beta-catenin stain**
- **Supplementary Figure 2: p40 stain**
- **Supplementary Figure 3: pan-keratin stain**
- **Supplementary Figure 4: SMA stain**
- **Supplementary Figure 5: t-SNE Bethesda classifier v31**
- **Supplementary Figure 6: UMAP Bethesda classifier**



Copyright: © 2025 The author(s). This is an open access article distributed under the terms of the Creative Commons Attribution 4.0 International License (https://creativecommons.org/licenses/by/4.0/), which permits unrestricted use, distribution, and reproduction in any medium, provided the original author and source are credited, a link to the Creative Commons license is provided, and any changes are indicated. The Creative Commons Public Domain Dedication waiver (https://creativecommons.org/publicdomain/zero/1.0/) applies to the data made available in this article, unless otherwise stated.

DOI: https://doi.org/10.17879/freeneuropathology-2025-9012


## Supplementary Figure 1: Beta-catenin stain

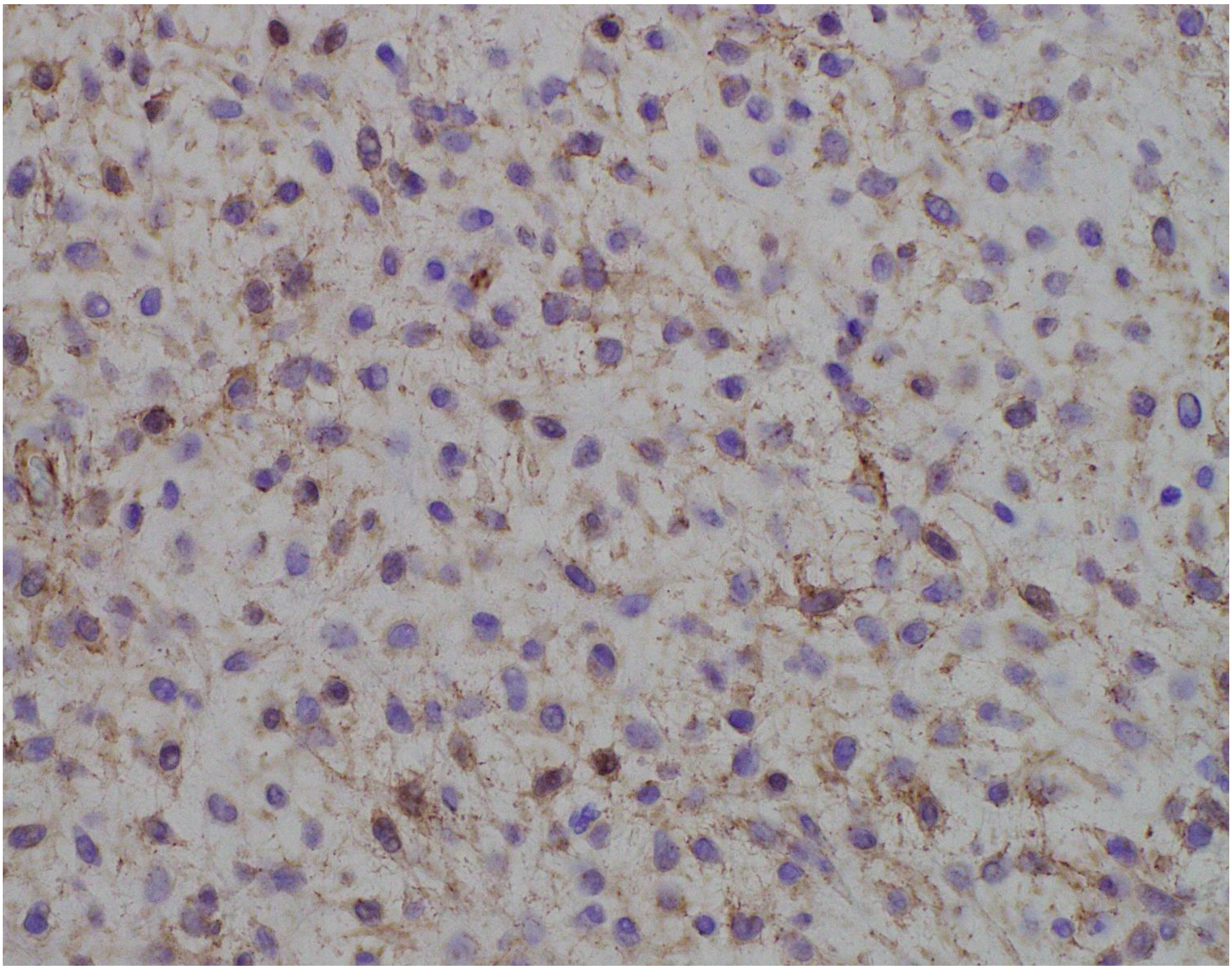

freeneuropathology.org

Copyright: © 2025 The author(s). This is an open access article distributed under the terms of the Creative Commons Attribution 4.0 International License (https://creativecommons.org/licenses/by/4.0/), which permits unrestricted use, distribution, and reproduction in any medium, provided the original author and source are credited, a link to the Creative Commons license is provided, and any changes are indicated. The Creative Commons Public Domain Dedication waiver (https://creativecommons.org/publicdomain/zero/1.0/) applies to the data made available in this article, unless otherwise stated.

## Supplementary Figure 2: p40 stain

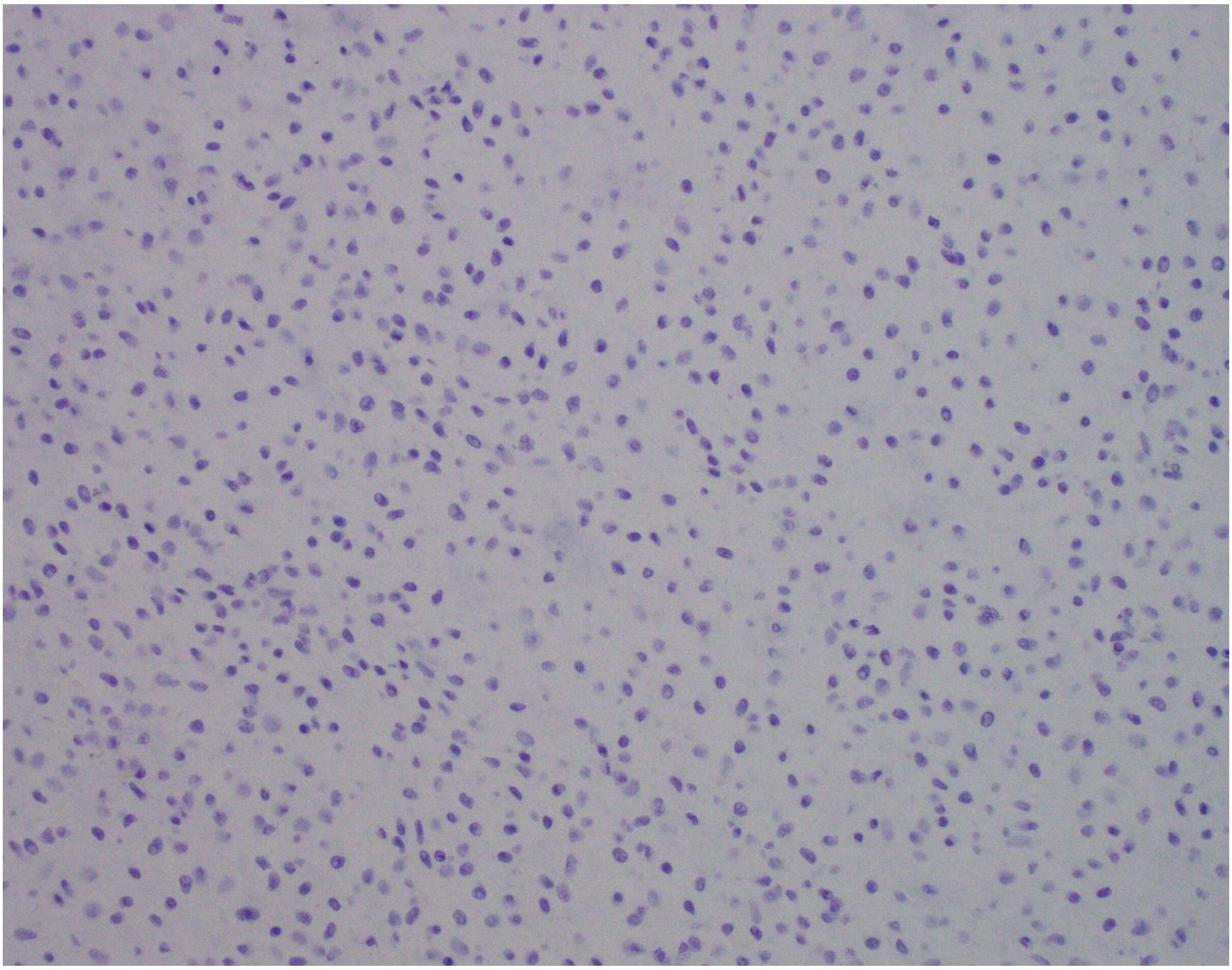

Copyright: © 2025 The author(s). This is an open access article distributed under the terms of the Creative Commons Attribution 4.0 International License (https://creativecommons.org/licenses/by/4.0/), which permits unrestricted use, distribution, and reproduction in any medium, provided the original author and source are credited, a link to the Creative Commons license is provided, and any changes are indicated. The Creative Commons Public Domain Dedication waiver (https://creativecommons.org/publicdomain/zero/1.0/) applies to the data made available in this article, unless otherwise stated.

## Supplementary Figure 3: pan-keratin stain

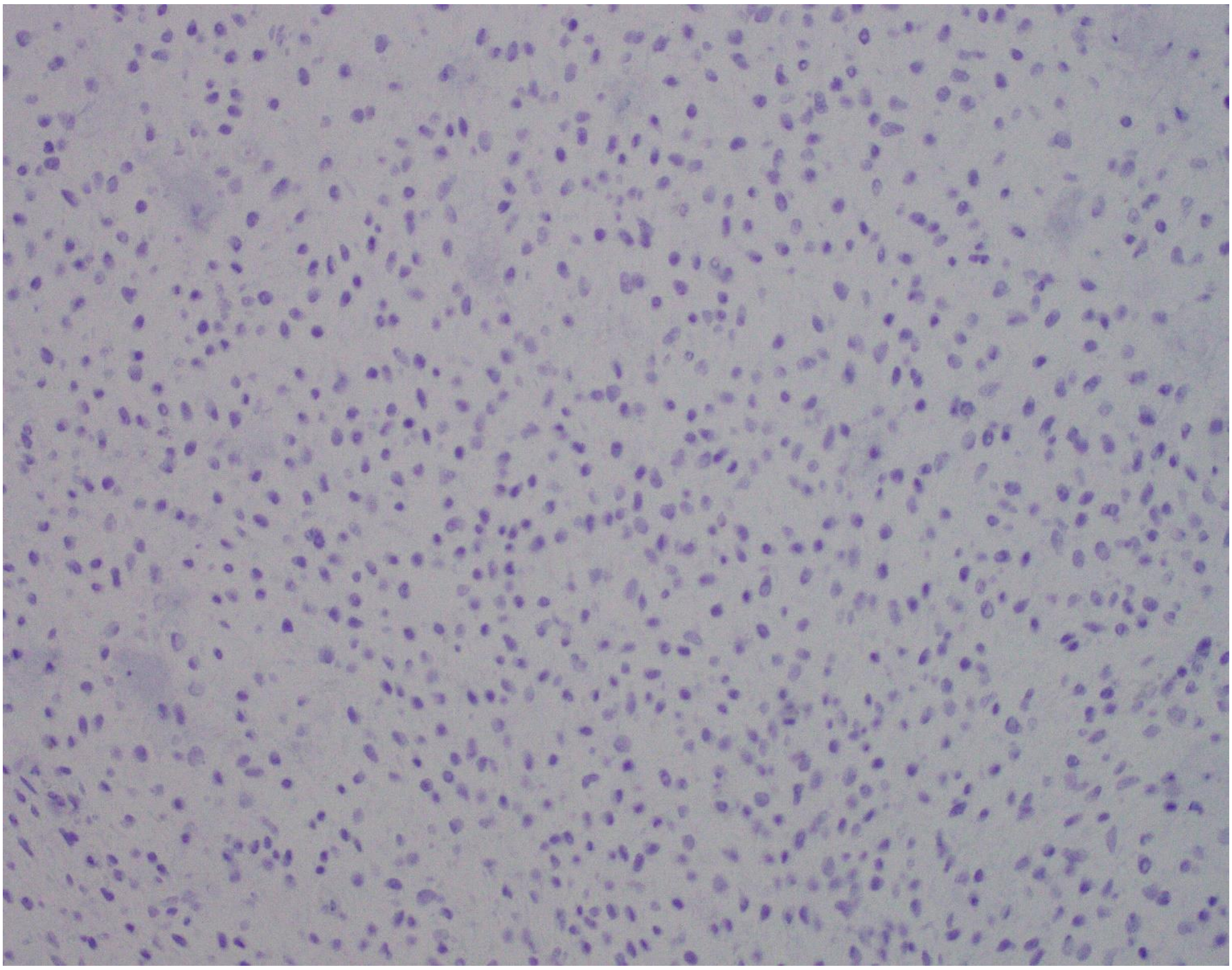

freeneuropathology.org

Copyright: © 2025 The author(s). This is an open access article distributed under the terms of the Creative Commons Attribution 4.0 International License (https://creativecommons.org/licenses/by/4.0/), which permits unrestricted use, distribution, and reproduction in any medium, provided the original author and source are credited, a link to the Creative Commons license is provided, and any changes are indicated. The Creative Commons Public Domain Dedication waiver (https://creativecommons.org/publicdomain/zero/1.0/) applies to the data made available in this article, unless otherwise stated.

## Supplementary Figure 4: SMA stain

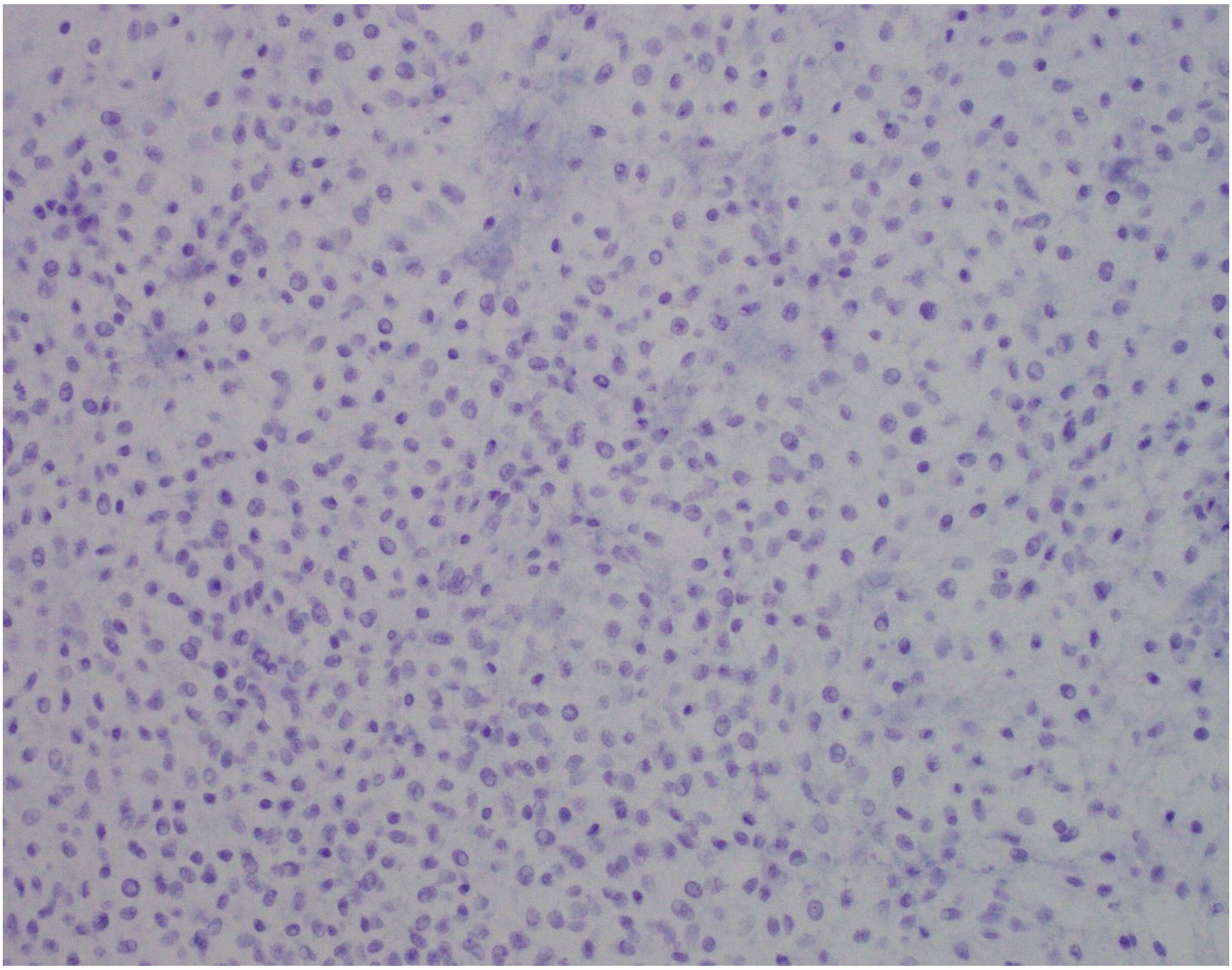



Copyright: © 2025 The author(s). This is an open access article distributed under the terms of the Creative Commons Attribution 4.0 International License (https://creativecommons.org/licenses/by/4.0/), which permits unrestricted use, distribution, and reproduction in any medium, provided the original author and source are credited, a link to the Creative Commons license is provided, and any changes are indicated. The Creative Commons Public Domain Dedication waiver (https://creativecommons.org/publicdomain/zero/1.0/) applies to the data made available in this article, unless otherwise stated.

DOI: https://doi.org/10.17879/freeneuropathology-2025-9012


## Supplementary Figure 5: t-SNE Bethesda classifier v3

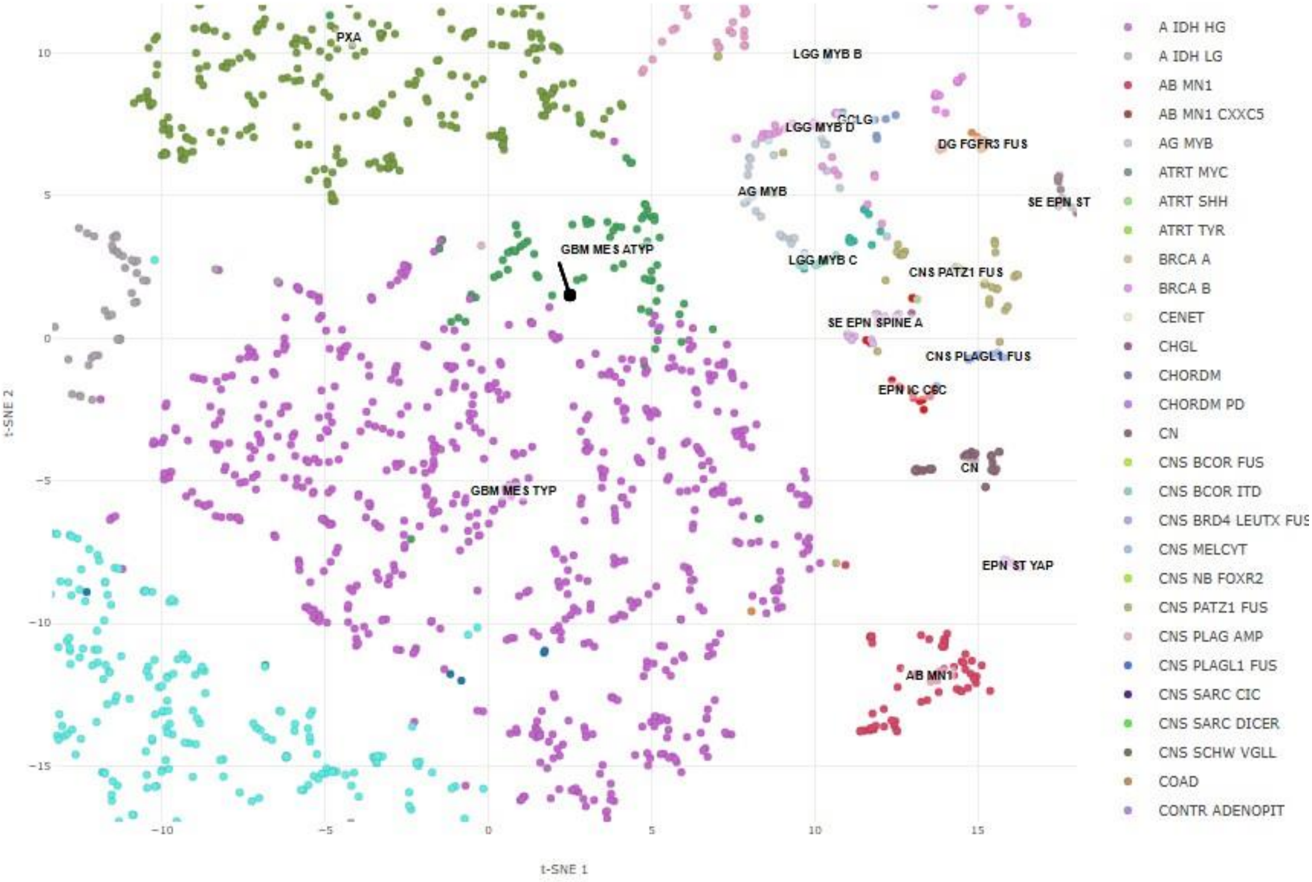


freeneuropathology.org

Copyright: © 2025 The author(s). This is an open access article distributed under the terms of the Creative Commons Attribution 4.0 International License (https://creativecommons.org/licenses/by/4.0/), which permits unrestricted use, distribution, and reproduction in any medium, provided the original author and source are credited, a link to the Creative Commons license is provided, and any changes are indicated. The Creative Commons Public Domain Dedication waiver (https://creativecommons.org/publicdomain/zero/1.0/) applies to the data made available in this article, unless otherwise stated.

Free Neuropathology 6:21 (2025)
DOI: https://doi.org/10.17879/freeneuropathology-2025-9012


## Supplementary Figure 6: UMAP Bethesda classifier

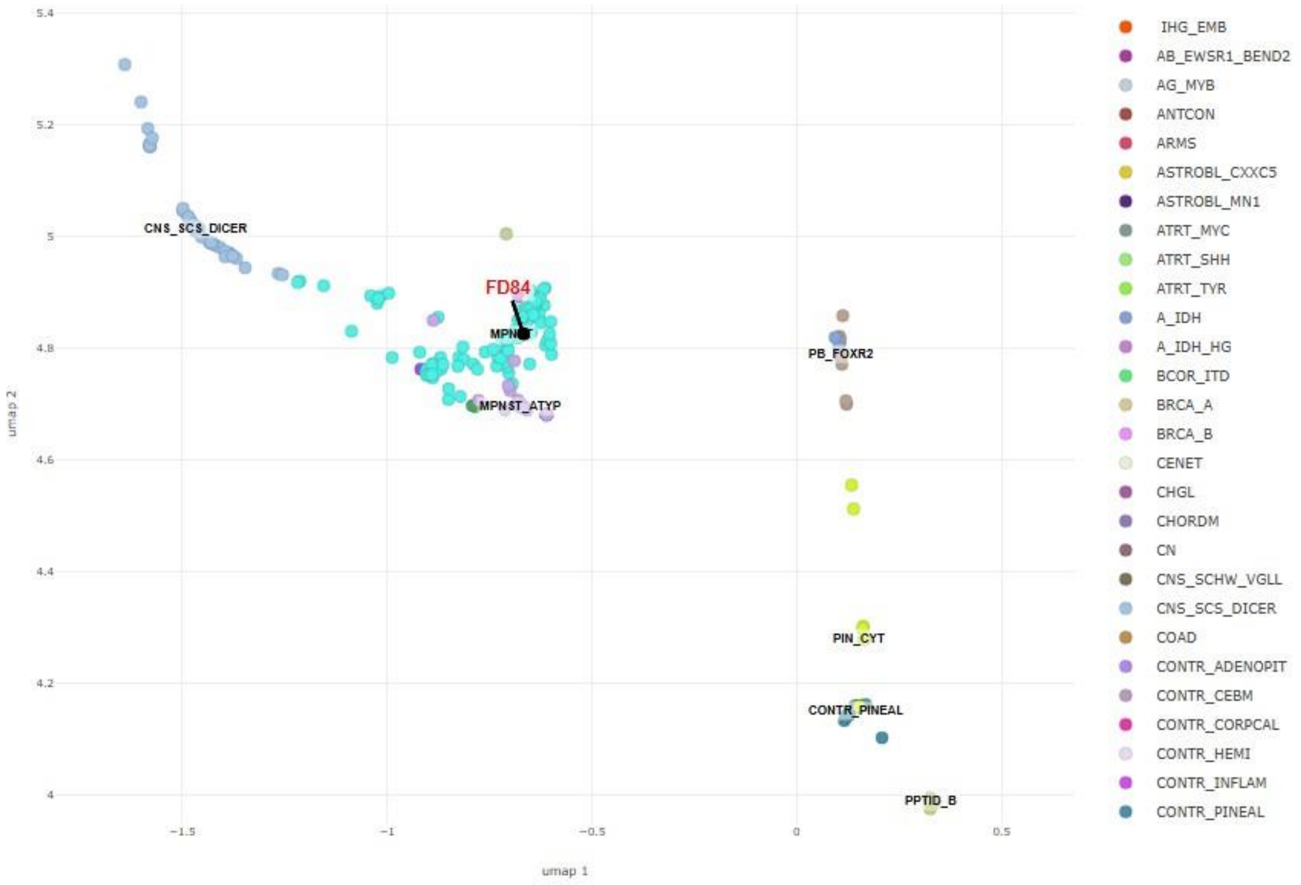


freeneuropathology.org

Copyright: © 2025 The author(s). This is an open access article distributed under the terms of the Creative Commons Attribution 4.0 International License (https://creativecommons.org/licenses/by/4.0/), which permits unrestricted use, distribution, and reproduction in any medium, provided the original author and source are credited, a link to the Creative Commons license is provided, and any changes are indicated. The Creative Commons Public Domain Dedication waiver (https://creativecommons.org/publicdomain/zero/1.0/) applies to the data made available in this article, unless otherwise stated.
